# Postponed healthcare in The Netherlands during the COVID-19 pandemic and its impact on self-reported health

**DOI:** 10.3389/frhs.2023.1181532

**Published:** 2023-06-23

**Authors:** Kirsten Visscher, Lisanne H. J. A. Kouwenberg, Marije Oosterhoff, Adriënne H. Rotteveel, G. Ardine de Wit

**Affiliations:** ^1^ Centre for Nutrition, Prevention and Healthcare, National Institute for Public Health and the Environment, Bilthoven, Netherlands; ^2^ Department of Public and Occupational Health, Amsterdam UMC Location University of Amsterdam, Amsterdam, Netherlands; ^3^ Faculty of Science, Department of Health Sciences and Amsterdam Public Health Research Institute, Vrije Universiteit Amsterdam, Amsterdam, Netherlands

**Keywords:** COVID-19, postponement of healthcare, survey, health effects, primary care, hospital care, dental care, paramedical care

## Abstract

**Background:**

Healthcare services have been seriously disrupted during the COVID-19 pandemic. The aim of this study was to examine the extent to which Dutch citizens have experienced postponed healthcare and how this affected their self-reported health. In addition, individual characteristics that were associated with experiencing postponed healthcare and with self-reported negative health effects were investigated.

**Methods:**

An online survey about postponed healthcare and its consequences was developed, and sent out to participants of the Dutch LISS (Longitudinal Internet Studies for the Social Sciences) panel (*n* = 2.043). Data were collected in August 2022. Multivariable logistic regression analyses were carried out to explore characteristics associated with postponed care and self-reported negative health outcomes.

**Results:**

Of the total population surveyed, 31% of the panel experienced postponed healthcare, either initiated by the healthcare provider (14%), on their own initiative (12%) or as a combination of both (5%). Postponed healthcare was associated with being female (OR = 1.61; 95% CI = 1.32; 1.96), presence of chronic diseases (OR = 1.55, 95% CI = 1.24; 1.95), high income (OR = 0.62, 95% CI = 0.48; 0.80) and worse self-reported health (poor vs. excellent OR = 2.88, 95% CI = 1.17; 7.11). Overall, 40% experienced temporary or permanent self-reported negative health effects due to postponed care. Negative health effects as a result of postponed care were associated with presence of chronic conditions and low income levels (*p* < 0.05). More respondents with worse self-reported health and foregone healthcare reported permanent health effects as compared to those with temporary health effects (*p* < 0.05).

**Discussion:**

People with an impaired health status are most likely to experiencing postponed healthcare and negative health consequences as a result. Furthermore, those with negative health consequences decided to forego health by themselves more often. As part of long-term plans to maintain the accessibility of healthcare services, specific attention should be paid to reaching out to people with an impaired health status.

## 1. Introduction

The COVID-19 pandemic has caused unprecedented challenges for numerous industries, including healthcare systems worldwide (1). Many countries faced hurdles in utilization of, and access to healthcare during the pandemic. These include for example reduction in services offered by healthcare professionals, and barriers to seek for medical care by patients due to fear (2). The first COVID-19 patients in The Netherlands were identified in February 2020 (3). In March 2020 the Dutch government announced the first measures in an attempt to slow down the spread of the virus and protect the most vulnerable people. A partial lockdown was announced, restaurants, schools and daycare centers closed and people were advised to work from home. However, these measures could not prevent the healthcare system from being largely disrupted. During the pandemic, regular healthcare services worldwide were canceled or postponed by healthcare providers (2, 4, 5). This was among other reasons due to the fact that regular capacity of healthcare services was shifted to COVID-19 patients. In addition, regular healthcare contacts decreased because people were afraid to get infected by visiting their GP or the hospital, or they had other reasons to postpone or cancel the healthcare they needed (6–10). These reductions in healthcare provision and healthcare demand resulted in large decreases in the number of appointments and treatments during the pandemic, affecting many citizens (11–14). For instance, a study among older adults (aged between 62 and 102 years old) in The Netherlands found that 35% of the study participants experienced postponed healthcare (15). Research among Austrian adults found that 20% of citizens reported unmet healthcare needs in 2020 (9). Some studies found that people with chronic diseases and poor health had to deal more often with postponed healthcare (15, 16). An online survey among US citizens reported that 20% of households experienced postponed healthcare, and that 57% of those reported negative health consequences (11). Another online survey set out among US citizens reported that 36% of adults were exposed to postponed health care. Approximately one-third of this group reported that this resulted in negative health outcomes (5).

In The Netherlands, the disruption in healthcare services resulted in a significant loss of healthy life years of patients that had to wait for elective surgery (17, 18). In 2020 and 2021, an average of 1.05 quality-adjusted life years (QALYs) was estimated to be lost for each elective surgery that was postponed, with a range of 0.01–6.45 QALYs per case. Less is known about the consequences of postponed healthcare from the perspective of citizens in the Netherlands, an affluent country with universal health insurance and access to healthcare for all (19). Therefore this study focuses on postponed healthcare and its consequences for self-reported health among citizens in The Netherlands. In this paper, the term “postponed healthcare” is used to describe both postponed and cancelled healthcare as initiated by the patient and/or the healthcare provider. Postponed healthcare initiated by the patient refers to delayed or cancelled health seeking. For example, this could include cancelling an appointment with a specialist in the hospital, or not making an appointment with the general practitioner because the patient is afraid of contracting a COVID-19 infection in the doctor's office. Postponed healthcare by the healthcare provider could for example include a cancelled appointment at the specialists' outpatient clinic, or not being able to make an appointment with a healthcare professional when deemed necessary by the patient. Self-reported health expresses the subjective assessment by the respondent of his or her current health status. This concept is often operationalized using a five-item Likert scale and can be used as a general indicator of the health status of (a subgroup of) the population (20). The aim of this study is threefold: (1) to describe the extent of postponement of healthcare during the COVID-19 pandemic; (2) to describe the self-reported short- and long term health impact of postponed healthcare and (3) to study what characteristics are associated with postponed healthcare and negative health effects.

## 2. Materials and methods

### 2.1. Research design

A cross-sectional web survey was administered under a representative group of Dutch citizens. One questionnaire was developed for the purpose of this study. The data from this questionnaire was linked to the data from a previous questionnaire set out among the same sample.

### 2.2. Study population

Respondents were recruited using the LISS (Longitudinal Internet studies for the Social Sciences) panel. The LISS panel, administered by Centerdata (Tilburg University, The Netherlands), is a Dutch panel where respondents are randomly selected based on a sample of households drawn from the population register by Statistics Netherlands (21). Therefore, every household in the Netherlands has an equal probably of being invited for the LISS panel, with the prerequisite that the overall sample reflects the countrywide distribution of socio-economic status. It is not possible to self-register for the panel. Respondents receive compensation for completing surveys, and if they do not have access to internet or a device to fill in the surveys, these are provided free of charge. Respondents participating in the LISS panel can choose themselves which and how many surveys they complete each month.

### 2.3. Sampling and sample size

The total LISS panel consists of 5,000 households, comprising approximately 7,500 individuals. Part of these individuals (*n* = 2,540) filled in the “Health Core Study” in November 2021. This survey included a broad range of questions about the health status of the respondent, including whether they are suffering from chronic conditions and self-reported health. The respondents who filled in this survey were invited to partake in the survey about delayed healthcare which was designed specifically for this study. This part of the data was collected in August 2022. We intended to include approximately 2,000 respondents representative for the Dutch society in terms of age, education and region. To fill the strata, the research agency used the method of purposive sampling. To ensure the net sample is representative, younger panel members were oversampled as they tend to have a lower response rate. This resulted in a sample size of 2,043 respondents for this study.

### 2.4. Data collection

A web survey was developed concerning experiences with postponed healthcare between March 2020 (first full month with COVID-19 infections in The Netherlands) and the time of data collection (August 2022). Information on self-reported background characteristics of respondents (e.g., age, sex, education level, income, self-reported health), was available from previously collected data in other surveys of the LISS panel. The questions of the web survey are shown in Supplementary Appendix S1. Each respondent was asked whether their healthcare appointments had been postponed or cancelled by the healthcare provider and/or whether and why they had foregone healthcare or had postponed healthcare on their own initiative. Follow-up questions were presented for both sections: (a) postponement by the healthcare provider, and (b) postponement on the respondent's own initiative. Respondents indicated from a list of healthcare providers of various disciplines which healthcare provider was involved (general practitioner, dentist, physical therapist, dietitian, or occupational therapist, specialist doctor in a hospital, home care, psychologist or psychiatrist, or other). For the selected healthcare providers follow-up questions were asked, for instance about the frequency of postponed appointments (1, 2 or 3+ times), about the self-perceived temporary and permanent negative health consequences due to postponed healthcare with this healthcare provider (yes a lot, yes a bit, none) and about whether the respondent received catch-up care for the postponed appointment. If more than two healthcare providers were selected in each section, the follow-up questions were asked for a random selection of two types of healthcare providers to prevent excessive survey length. Respondents were also asked to rate statements about, amongst others, temporary or permanent deterioration of health (“due to postponed healthcare during the COVID-19 pandemic, my health temporarily got worse”, “due to postponed healthcare during the COVID-19 pandemic, my health got permanently worse”). Response categories were “completely disagree”, “disagree”, “neutral”, “agree” or “completely agree”, including an opt-out possibility (see Supplementary Appendix S1).

### 2.5. Outcomes

Outcomes were: (1) experience with postponement of healthcare; and (2) self-reported negative health consequences. Respondents were categorized into two groups: respondents who experienced postponed healthcare at any point in time and respondents who did not. The number of healthcare providers that was involved in postponed healthcare was counted separately for postponed healthcare by the provider and for postponed healthcare on the initiative of the respondent. Additionally (descriptive analysis only), the total number of ticked healthcare providers was multiplied by the frequency of postponed contact moments indicated for each provider. This can be seen as a minimum total frequency of postponed contact moments, as (1) the frequency was asked for a maximum of two random healthcare providers (contact frequency for additional healthcare providers was set at 1), and (2) the contact frequency was maximized at 3 for the first two healthcare providers. Temporary or permanent health consequences were classified as negative health consequences in one or more follow-up questions for each healthcare provider (for those who chose response options: yes a little, yes a lot) or scoring 4–5 points on the Likert scale for the general statement about temporary or permanent health consequences (for those who chose response options: agree and completely agree), respectively.

### 2.6. Covariates

Background characteristics of respondents were obtained from previously collected data from the Health Core Study 2021 of the LISS panel. The following covariates were included in the analysis: income (personal gross income), living in an urban environment, presence and number of chronic conditions (suffering from any kind of long-standing disease, affliction, handicap) and self-reported health during the pandemic (poor, moderate, good, very good, excellent). Living in an urban environment was defined as strongly or extremely urbanized (yes/no) on a five-point scale for the urbanicity of the place of residence (not, hardly, moderate, strongly, and extremely urbanized). This cut-off point was chosen because the Netherlands is a highly urbanized country.

### 2.7. Statistical analyses

The data of the Health Core Study 2021 was linked to the data collected from the survey designed for this study by making use of the unique ID-number of the respondents. Univariate differences in background characteristics between groups were assessed using two-tailed independent *t*-tests for continuous variables, and Pearson's *χ*^2^ test for categorical variables. The following groups were assessed: respondents with vs. without postponed healthcare, respondents with vs. without negative health consequences, and respondents with temporary vs. permanent health consequences. Multivariable logistic regression with backward elimination (likelihood ratio test, *p* = 0.05) was used. Multicollinearity among the variables was checked by the tolerance and variance inflation factor (VIF). Due to small numbers, no stratified analyses for initiative (initiative of the healthcare provider or own initiative) or healthcare providers were performed. To explore any differences between forgone healthcare initiated by the respondent and postponed healthcare on the initiative of healthcare providers, a sensitivity analyses was performed among the subgroup who did not experience postponed dental care, as this was most often reported by respondents who had foregone healthcare by themselves. For a subset of the sample (*n* = 1,634) information on self-reported health before the pandemic was available. In a sensitivity analysis, the analyses were repeated with self-reported health before the pandemic instead of self-reported health during the pandemic to assess potentially causal relationships between self-reported health and the outcomes. Statistical analyses were performed using Stata version 17 (StataCorp. 2021. Stata Statistical Software: Release 17. College Station, TX: StataCorp LLC.).

## 3. Results

The response rate was 80.4% (*n* = 2,043 respondents out of 2,540 invited panel members). The study sample was representative for the Dutch population in terms of age, sex and education level (see Supplementary Appendix S2). Respondents with incomplete data on covariates (e.g., income level, urbanicity) had a lower educational level (*p* < 0.05) as compared to respondents with complete data, but information on other covariates was comparable (e.g., age, self-reported sex, chronic diseases, self-reported health). The first column of Table 1 shows descriptive statistics of the full sample (*n* = 2,043). The average age of the study population was 48.8 years old (range: 16–95 years), 54.7% was female and 29.7% reported having one or multiple chronic disease(s).

**Table 1:** Background characteristics of the total sample (*n* = 2,043), and characteristics of respondents who experienced postponed healthcare or not.

		Total sample (*n* = 2,043		Postponed healthcare (*n* = 629) (30.8%)		No postponed healthcare (*n* = 1,414) (69.2%)		*p*
Age in years (*n* = 2, 043) (mean, sd)		48.80	(18.7)	47.88	(17.59)	49.22	(19.18)	0.136
Self-reported sex (*n* = 2,043) (*n*, %)	Female	1,118	(54.7)	395	(62.8)	723	(51.1)	0.000
	Male	925	(45.3)	234	(37.2)	691	(48.9)	
Education (*n* = 2, 043) (*n*, %)	Primary education	145	(7.1)	44	(7.0)	101	(7.1)	0.621
	Secondary education	590	(28.9)	169	(26.9)	421	(29.8)	
	Secondary vocational education	499	(24.4)	152	(24.2)	347	(24.5)	
	Higher professional education	518	(25.4)	168	(26.7)	350	(24.8)	
	University education	291	(14.2)	96	(15.3)	195	(13.8)	
Living in an urban environment (*n* = 2,041) (*n*, %)		1,021	(50.0)	327	(52%)	694	(49%)	0.218
Presence of chronic disease(s) (*n* = 2,043) (*n*, %)		607	(29.7)	247	(39.3)	360	(25.5)	0.000
Self-reported health during the pandemic (*n* = 2,043) (*n*, %)	Poor	30	(1.5)	14	(2.2)	16	(1.1)	0.000
	Moderate	292	(14.3)	129	(20.5)	163	(11.5)	
	Good	1,163	(56.9)	348	(55.3)	815	(57.6)	
	Very good	456	(22.3)	118	(18.8)	338	(23.9)	
	Excellent	102	(5.0)	20	(3.2)	82	(5.8)	
Income in € (*n* = 1,923) (*n*, %)	€0–€1.249	480	(25.0)	156	(26.5)	324	(24.3)	0.798
	€1.250–€2.399	469	(24.4)	130	(22.1)	339	(25.4)	
	€2.400–€3.499	473	(24.6)	147	(25.0)	326	(24.4)	
	≥€3.500	501	(26.0)	155	(26.4)	346	(25.9)	

*n*, number; sd, standard deviation.

### 3.1. Postponed healthcare

The second and third column of Table 1 report the background characteristics of the sample, subdivided into whether or not respondents experienced postponed healthcare. Among the 2,043 respondents, 629 respondents (31%) reported to have experienced postponed healthcare. More specifically, 277 (14%) respondents experienced postponement caused by a healthcare provider, 245 (12%) respondents only postponed healthcare on their own initiative, and the remaining 107 (5%) respondents experienced both postponement by the healthcare providers and postponed healthcare themselves. Female respondents and respondents with an impaired health status (chronic condition present and/or a moderate or poor self-reported health status) reported more often that they experienced postponed healthcare than male respondents or respondents with better health (*p* < 0.05). The reasons for respondents to postpone or forgo healthcare are presented in Supplementary Appendix S3. The fear of being infected with the coronavirus was most often mentioned as reason to postpone or forgo healthcare (Supplementary Appendix S3). Supplementary Appendix S4 shows delayed care divided by type of healthcare provider.

### 3.2. Characteristics associated with postponed healthcare

Female sex [odds ratio (OR) = 1.61], chronic conditions (OR = 1.55), and self-reported health (OR poor vs. excellent = 2.88) were positively related to experiencing postponed healthcare (Table 2). A low education level (OR low vs. high = 0.62) was associated with less postponed healthcare. No other associations between covariates and postponed healthcare were statistically significant. In the sensitivity analyses, a model for the subgroup without postponed dental care and a model including self-reported health before instead of during the pandemic, showed comparable patterns (Supplementary Appendix S5 and Supplementary Table A5.1 and Supplementary Appendix S6 and Supplementary Table A6.1). This sensitivity analyses were carried out to explore potentially causal relationships.

**Table 2:** Logistic regression of experiencing postponed healthcare.

Variable	Category	Full model^a^		Final model^b^	
		OR (95% CI)	*p*	OR (95% CI)	*p*
Age	15–24 years	ref			
	25–34 years	1.09 (0.71–1.67)	0.70		
	35–44 years	1.43 (0.93–2.20)	0.10		
	45–54 years	0.87 (1.57–1.33)	0.53		
	55–64 years	0.88 (0.58–1.34)	0.55		
	65 years and older	0.73 (0.49–1.09)	0.12		
Self-reported sex	Female	1.61* (1.30–2.00)	0.00	1.61* (1.32–1.96)	0.00
	Male	ref		ref	
Education level	Low	0.72* (0.52–0.98)	0.04	0.62* (0.48–0.80)	0.00
	Intermediate	1.04 (0.81–1.32)	0.78	0.91 (0.73–1.14)	0.42
	High	ref		ref	
Income in €	€0–€1.249	1.01 (0.71–1.44)	0.94		
	€1.250–€2.399	0.76 (0.55–1.05)	0.09		
	€2.400–€3.499	0.91 (0.68–1.22)	0.52		
	≥€3.500	ref			
Urbanicity level	Urbanized	1.15 (0.94–1.40)	0.18		
	Not urbanized	ref			
Chronic disease	Yes	1.76* (1.39–2.24)	0.00	1.55* (1.24–1.95)	0.00
	No	ref		ref	
Self-reported health during the pandemic	Poor	2.67* (1.06–6.71)	0.04	2.88* (1.17–7.11)	0.02
	Moderate	3.16* (1.74–5.73)	0.00	2.50* (1.41–4.42)	0.00
	Good	1.86* (1.09–3.17)	0.02	1.56 (0.93–2.61)	0.09
	Very good	1.59 (0.91–2.77)	0.10	1.33 (0.78–2.28)	0.29
	Excellent	ref		ref	

CI, confidence interval; n, number; OR, odds ratio; ref, reference category.

^a^ *n* = 1,921, constant: beta = 0.17, tolerance >0.7 and VIF < 1.4.

^b^ *n* = 2,043, constant: beta = 0.21, tolerance >0.8 and VIF < 1.3.

* *p* < 0.05.

### 3.3. Negative health consequences

From all respondents who experienced postponed healthcare (*n* = 629), 40% reported any temporary and/or permanent negative consequences for their health (Table 3). A permanent negative health impact was reported by 19% of the respondents who experienced some form of postponed healthcare (Table 4), equaling 2.4% (0.31 × 0.4 × 0.19) of the whole sample. Of all respondents who received (partial) catch-up care (*n* = 537), 17% still reported permanent health consequences (not shown). Of the respondents whose healthcare had not been caught up (*n* = 92), 32% reported permanent health consequences (not shown).

**Table 3:** Characteristics of respondents who experienced either or not negative consequences of postponed healthcare.

		No health consequences (*n* = 377; 59.9%)		Any health consequences (*n* = 252; 40.1%)		*p*	Only temporary consequences (*n* = 133; 21.1%)^a^		Permanent consequences (*n* = 119; 18.9%)^a^		*p*
Age in years (*n* = 629) (mean, sd)		49.1	(17.6)	46.1	(17.5)	.036	44.4	(17.6)	48.0	(17.2)	.012
Self-reported sex (*n* = 629) (*n*, %)	Female	144	(61.8)	162	(64.3)	.398	89	(66.9)	73	(61.3)	0.357
	Male	233	(38.2)	90	(35.7)		44	(33.1)	46	(38.7)	
Education (*n* = 629) (*n*, %)	Primary education	25	(6.6)	19	(7.5)	.137	9	(6.8)	10	(8.4)	0.971
	Secondary education	95	(25.2)	74	(29.4)		38	(28.6)	36	(30.3)	
	Secondary vocational education	83	(22.0)	69	(27.4)		38	(28.6)	31	(26.1)	
	Higher professional education	112	(29.7)	56	(22.2)		31	(23.3)	25	(21.0)	
	University education	62	(16.5)	34	(13.5)		17	(12.8)	17	(14.3)	
Living in an urban environment (*n* = 628) (*n*, %)		196	(52.1)	131	(52.0)	.972	65	(48.9)	66	(55.5)	.296
Presence of chronic disease (*n* = 629) (*n*, %)		129	(34.2)	118	(46.8)	.002	59	(44.4)	59	(49.6)	.407
Self-reported health during the pandemic (*n* = 629) (*n*, %)	Poor	4	(1.0)	10	(4.0)	.009	2	(1.5)	8	(6.7)	.004
	Moderate	67	(17.8)	62	(24.6)		24	(18.1)	38	(31.9)	
	Good	214	(56.8	134	(53.2)		83	(62.4)	51	(42.9)	
	Very good	81	(21.5)	37	(14.7)		21	(15.8)	16	(13.5)	
	Excellent	11	(2.9)	9	(3.6)		3	(2.3)	6	(5.0)	
Income in € (*n* = 588) (*n*, %)	€0–€1.249	80	(22.7)	76	(32.3)	.008	40	(31.3)	36	(33.6)	.798
	€1.250–€2.399	76	(21.5)	54	(23.0)		28	(21.9)	26	(24.3)	
	€2.400–€3.499	88	(24.9)	59	(25.1)		32	(25.0)	27	(25.2)	
	≥€3.500	109	(30.9)	46	(19.6)		28	(21.9)	18	(16.8)	
Number of selected healthcare providers (*n* = 629): postponed by healthcare provider (mean, sd)		0.6	(0.6)	1.1	(0.9)	.000	1.2	(0.9)	0.9	(1.0)	.014
Number of appointments that were postponed by healthcare provider (*n* = 629) (mean, sd)		0.8	(1.0)	1.9	(1.8)	.000	2.0	(1.6)	1.8	(2.0)	.291
Number of selected healthcare providers (*n* = 629): postponed by respondent (mean, sd)		0.7	(0.7)	0.9	(0.9)	.000	0.6	(0.8)	1.1	(1.0)	.000
Number of appointments that were postponed by respondent (*n* = 629) (mean, sd)		0.9	(1.0)	1.4	(1.6)	.000	0.8	(1.2)	2.0	(1.9)	.000
Postponed by (*n* = 629) (*n* %)	Only healthcare provider	165	(43.8)	112	(44.4)	.000	75	(56.4)	37	(31.1)	.000
	Both by healthcare provider and respondent themselves	39	(10.3)	68	(27.0)		30	(22.6)	38	(31.9)	
	Only by respondent	173	(45.9)	72	(28.6)		28	(21.1)	44	(37.0)	
Appointments (partially) caught-up (*n* = 629) (*n*, %)		322	(85.4)	215	(85.3)	.974	125	(94.0)	90	(75.6)	.000

*n,* number; sd, standard deviation.

^a^ Of 629 respondents.

**Table 4:** Logistic regression of experiencing either or not negative health consequences of postponed healthcare.

Variable	Category	Full model^a^		Final model^b^	
		OR (95% CI)	*p*	OR (95% CI)	*p*
Age	15–24 years	ref		ref	
	25–34 years	2.65* (1.28–5.49)	0.01	1.97* (1.04–3.73)	0.04
	35–44 years	1.92 (0.91–4.02)	0.09	1.53 (0.81–2.89)	0.20
	45–54 years	1.19 (0.56–2.55)	0.65	0.90 (0.48–1.70)	0.74
	55–64 years	0.67 (0.33–1.37)	0.27	0.59 (0.31–1.10)	0.10
	65 years and older	0.88 (0.43–1.79)	0.72	0.67 (0.36–1.24)	0.20
Self-reported sex	Female	0.85 (0.58–1.25)	0.40		
	Male	ref			
Education level	Low	1.41 (0.79–2.53)	0.25	1.98* (1.23–3.19)	
	Intermediate	1.51 (0.97–2.34)	0.07	1.79* (1.22–2.65)	
	High	ref		ref	
Urbanicity level	Urbanized	0.94 (0.66–1.33)	0.72		
	Not urbanized	ref			
Income	€0–€1.249	2.38* (1.26–4.48)	0.01		
	€1.250–€2.399	1.37 (0.78–2.41)	0.28		
	€2.400–€3.499	1.29 (0.76–2.20)	0.34		
	≥€3.500	ref			
Chronic disease	Yes	1.92* (1.26–2.91)	0.00	2.14* (1.48–3.09)	0.00
	No	ref		ref	
Self-reported health during the pandemic	Poor	1.27 (0.24–6.87)	0.78		
	Moderate	0.91 (0.30–2.75)	0.87		
	Good	0.83 (0.30–2.35)	0.73		
	Very good	0.56 (0.19–1.64)	0.29		
	Excellent	ref			

^a^ *n* = 587, constant: beta = 0.33, tolerance >0.7 and VIF < 1.4.

^b^ *n* = 629, constant: beta = 0.34, tolerance >0.8 and VIF < 1.3.

*p < 0.05.

Table 3 shows the characteristics of respondents who experienced temporary or permanent negative consequences as a result of postponed healthcare. Younger respondents and respondents with worse health (presence of chronic disease, worse self-reported health status and lower self-rated health) reported more often negative consequences of postponed healthcare than respondents without negative health consequences (*p* < 0.05). Respondents who experienced negative health consequences had a higher number of postponed appointments compared to respondents with no health consequences (*p* < 0.05). Respondents who postponed or cancelled appointments on their own initiative reported less often any health consequences, compared to respondents who also experienced postponement by the healthcare provider (*p* < 0.05). Looking at respondents with permanent vs. temporary consequences, respondents with worse health and respondents who postponed or cancelled appointments on their own initiative reported more permanent health consequences (Table 3). Respondents who suffer from permanent consequences reported a higher number of appointments and a higher number of healthcare providers being involved in their postponed healthcare as compared to those with only temporary consequences. Respondents with permanent consequences reported less often that they had received catch-up care as compared to respondents with only temporary health consequences.

### 3.4. Characteristics associated with negative health consequences

Age group 25–34 years (vs. <25 years OR = 1.97), low education (OR low vs. high = 1.98) and presence of chronic conditions (OR = 2.14) were statistical significantly associated with negative health consequences (Table 4). In a sensitivity analysis, the model was run with self-reported health before the pandemic instead of during the pandemic to explore potentially causal relationships. This showed that presence of chronic diseases and worse self-reported health before the pandemic were associated with negative health consequences (Supplementary Appendix S5 and Supplementary Table A5.2). The model among the subgroup without postponed dental care showed a comparable pattern as presented in Table 4 (Supplementary Appendix S6 and Supplementary Table A6.1).

## 4. Discussion

This study aimed to describe the extent to which citizens have experienced postponed healthcare in the Netherlands during the COVID-19 pandemic, and to investigate the self-reported health impact of postponed healthcare. We also aimed to explore which patient characteristics were associated with postponed healthcare and negative health impact.

In this study we found that almost one third of a nationally representative sample of Dutch respondents experienced postponed healthcare, either on the initiative of the healthcare provider or on their own initiative. More females, respondents with chronic conditions and worse self-reported health reported postponed healthcare. From all respondents who experienced some form of postponed healthcare, 40% experienced negative health consequences and 19% of respondents had a permanent negative health impact, equaling 2.4% of the whole sample. Respondents with low education levels reported less often postponed healthcare, but reported more often negative health consequences. Presence of chronic diseases was associated with both postponed healthcare and with negative health consequences.

Other international studies found similar numbers for the frequency of postponed healthcare (5, 8, 15, 22). Some studies reported higher numbers (22), but focused specifically on medical care for serious health problems requiring hospital care, in contrast to our study which also included primary and dental care. Women were confronted more often with postponed healthcare services than men. This was also found by other authors (10, 15). Schuster et al. show that female older adults more often experienced healthcare-initiated cancellations compared to male older adults (15). Papautsky et al. (2021) also found that cisgender women were more likely to experience postponed healthcare, with gender identity being the most important predictor in their model on postponed healthcare (23). Low education levels were negatively associated with experiencing postponed healthcare, while negative health consequences were more often reported by respondents with low education levels. Various patterns have been found in the literature. Schmidt et al. (2022) did not find patterns between socio-economic variables and unmet needs during the pandemic, while Smolic et al. (2021) found that limited healthcare access was more common for the more educated (9, 16). It is therefore recommended to gain further insight into socioeconomic differences in postponed healthcare and adverse health effects across various countries. Our results also showed that respondents with worse health status or chronic diseases experienced more postponed healthcare. This finding is in accordance with other studies that reported that worse health or the presence of chronic conditions or disabilities increased the likelihood of experiencing postponements in healthcare delivery (5, 8, 15, 23). Reasons for this finding are unclear. Perhaps those with worse health status consume on average higher amounts of healthcare, which in turn makes it more likely that they were confronted with postponed healthcare. Another possibility is that people with an impaired health status experienced more often postponed healthcare because of their own concerns about getting infected with the coronavirus, or because of the perception of healthcare providers that the expected benefits of healthcare may not outweigh the risk of infection for the individual concerned. Indeed, fear of being infected by the coronavirus was quoted often as a reason to forego healthcare. Not only was worse health associated with postponement of healthcare, it was also associated with self-reported negative health consequences. In a sensitivity analysis, it was found that self-reported health before the pandemic was also associated with postponed healthcare and negative consequences. This might suggest that part of the associations between self-reported health and postponed healthcare and/or negative health consequences might be due to a worse health status before the pandemic. Due to small numbers, it was not possible to perform stratified analysis for the source of postponed healthcare (initiative of the provider or foregone healthcare on the initiative of citizens) and for different healthcare providers. A sensitivity analysis in a subgroup without postponed dental care showed similar patterns. More research is, however, recommended to explore whether the characteristics associated with postponed healthcare differ across different healthcare services.

A somewhat surprising finding is that those who postponed or cancelled appointments on their own initiative reported more often permanent negative health consequences. Other studies have also found associations between forgone healthcare and chronic diseases such as diabetes (24, 25). A possible explanation for this might be that those in poorer health postponed or cancelled their appointment because of their own concerns about getting infected by the coronavirus, and experienced negative health consequences as a result. This is also hypothesized by Werner and Tur-Sinai (2021) who mentioned that communication about the increased risk of COVID-morbidity and mortality for persons with diabetes might have led to fears of contracting COVID and forgone healthcare among those with chronic diseases (24).

People whose healthcare has not been (partially) caught-up reported more permanent negative consequences of postponement of healthcare compared to people that had already received some catch-up care. This result is in line with Gonzalez et al. (2021) who also showed that adults who had still not received the care at the time of the survey were more likely to report negative consequences compared to those who eventually received care but after a delay (5).

### 4.1. Implications

During the COVID-19 pandemic, regular healthcare services were not accessible for many citizens. The current study yields insight into the magnitude of this problem and described the characteristics of respondents for whom postponed healthcare was most prevalent and most impactful.

The Dutch government announced a long-term strategy for the future, should new episodes of the COVID-19 pandemic (or other epidemics or pandemics) occur (26). Maintaining access to healthcare services is a main goal in this strategy. Others also emphasized the need for equitable access of healthcare services (27, 28). The insights of this study may be helpful in detailing action plans for accessible healthcare in crisis situations. It was found that respondents with an impaired health status were most prone to postponed healthcare and negative health consequences, and frequently postponed or cancelled healthcare visits by themselves. This underlines the importance to reach out to those with an impaired health status in order to prevent permanent negative health consequences. It might also suggest that especially citizens with an impaired health status should be informed and stimulated to seek access to appropriate healthcare services when needed. Furthermore, it seems that those whose healthcare has been (partially) caught-up report fewer permanent negative health consequences. This highlights the importance of catching-up healthcare, which has not been an easy task so far. Waiting lists and backlogs have exploded in all countries (29). Identifying those who are in highest need of catch-up care, therefore remains of utmost importance.

### 4.2. Strengths and weaknesses

The current study provided insight into postponed healthcare in The Netherlands, including multiple health services. The response rate was high and the study sample was representative for the overall Dutch adult population. The survey was administered in August 2022, when the number of infections and hospitalizations was low for quite some time. This allowed respondents to reflect on the postponed healthcare and evaluate whether health-effects were temporary or more permanent. This study should also be interpreted in the light of several limitations. Self-reported data was collected, which may be subjected to recall bias resulting in over- or under-report of postponed healthcare, specifically for the beginning of the recall period. Stratified analysis for different types of healthcare and source of postponement (initiative of the provider or forgone healthcare) could not be performed due to relatively small numbers. Follow-up questions on postponed healthcare were also asked only for a maximum of two healthcare providers. We were therefore unable to perform detailed analyses for different types of healthcare providers. Finally, information on self-reported health before the pandemic was limited. Further research on the causality and directionality of health and negative health consequences due to postponed healthcare is recommended.

## 5. Conclusions

The findings of the current study suggest that people with an impaired health status health are most prone to experiencing postponed healthcare and negative health consequences as a result. Furthermore, they also decide to forego health by themselves. As part of long-term plans to maintain the accessibility of healthcare services, specific attention should be paid to reaching out to people with an impaired health status.

## Data Availability

The datasets presented in this article are not readily available because the data generated and analyzed in this study was obtained from LISS (Longitudinal Internet studies for the Social Sciences) panel, administered by Centerdata (Tilburg University, The Netherlands). The data is available upon request for scientific or policy related research. Requests to access the datasets should be directed to https://statements.centerdata.nl/Liss-panel-data-statement.
