# Supplementary material for: Postponed healthcare in The Netherlands during the COVID-19 pandemic and its impact on self-reported health
Source: Front Health Serv. 2023 Jun 23;3:1181532. doi: 10.3389/frhs.2023.1181532 (PMC10326615; doi:10.3389/frhs.2023.1181532)
Supplement: Supplementary file 1 [file Datasheet1.pdf]

Postponed medical care during the COVID-19 pandemic

Questionnaire administered to the LISS panel

**Date**	December 2022
**Version**	1.0
**Classification**	Standard

© Centerdata, Tilburg 2022

All rights reserved. No part of this publication may be reproduced, stored in a retrieval system, or transmitted, in any form or by any means, electronic, mechanical, photocopying, recording or otherwise, without the prior written permission of the publisher.

Table of contents

1	Project description	4
2	Introduction	5
3	Codebook	6

1 Project description

Title: Postponed medical care during the COVID-19 pandemic
Data file: vx22a
Funding source(s): ODISSEI; OCW (Domeinplan SSH); RIVM
Researcher(s): Kirsten Visscher (RIVM)
Project description: The aim of this study is to gather information about the number of people that had to deal with postponed medical care, the types of healthcare that were postponed, and whether this had consequences for one's health.
Sample: Participants of the most recent Health Core Study (November 2021). To make sure the net sample would be representative for the variables age, education and province, we oversampled younger panel members as they respond the least.
Overview of the response:

Selected number of household members:	2,540	(100.0%)
Non-response:	497	(19.6%)
Response:	2,043	(80.4%)
Complete:	2,039	(80.3%)
Incomplete:	4	(0.2%)

Date of data collection: August 2022

Titel: Uitgestelde zorg tijdens de coronapandemie
Databestand: vx22a
Financiering: ODISSEI; OCW (Domeinplan SSH); RIVM
Onderzoeker(s): Kirsten Visscher (RIVM)
Projectbeschrijving: Het doel van deze studie is informatie te verzamelen over de mate waarin men te maken kreeg met uitgestelde zorg, welke vorm van zorg is uitgesteld en of dit gevolgen had voor de gezondheid.
Steekproef: Deelnemers aan de meest recente afname van de Health Core Study (november 2021). Om ervoor te zorgen dat de netto steekproef representatief is op de verdeling naar leeftijd, opleiding en provincie, hebben we als uitgangspunt de jongere panelleden oververtegenwoordigd omdat deze groep het minst goed respondeert.
Responsoverzicht:

Selectie aantal leden huishouden:	2.540	(100,0%)
Nonrespons:	497	(19,6%)
Respons:	2.043	(80,4%)
Compleet:	2.039	(80,3%)
Incompleet:	4	(0,2%)

Datum van dataverzameling: augustus 2022

2 Introduction

In August 2022, the questionnaire "Uitgestelde zorg" (Postponed medical care) was fielded in the LISS panel[1].

The questionnaire was presented to 2,540 panel members and 2,039 respondents fully completed the survey (response percentage 80.3%).

[1] Reference to LISS panel data in text:
In this paper we make use of data of the LISS (Longitudinal Internet studies for the Social Sciences) panel administered by Centerdata (Tilburg University, The Netherlands).

3 Codebook

This codebook contains the questionnaire as administered to the LISS panel.

- Variable names: The variable names are printed in bold and correspond to the names in the dataset.
- The questionnaire routing is printed in bold and colored blue for each variable concerned.
  - = means "is equal to"
  - != means "is not equal to"
  - <= means "smaller or equal to"
  - >= means "larger or equal to"
- If there were buttons on a page for "I don't know, "I refuse to say" or "Not applicable", this will be indicated at the top at "Page" (when the button is clicked, the corresponding value is written to all variables that were present on the page)
- text: answer box (no limit to the length of the answer).
- string: answer box allowing a certain number of characters (standard is 255).
- empty: questions could be left unanswered
- Numerical variables: If ranges were used, these are printed in italics in the codebook if the respondent could not see them on the screen. If the respondent was able to see the ranges, the variables are printed in normal letter. Integer: If a question is not subject to any limit (integer), no range is indicated.
- 'Fills' (variable text) are given between straight brackets [].
- Variables in between curly brackets {} are not part of the dataset, but the corresponding questions or text were part of the questionnaire.

nomem_encr[2]
Number of the household member encrypted
integer

vx22a_m
Year and month of the field work period
integer

[2] Use nomem_encr as key variable for merging with other LISS panel studies from the LISS Panel Data Archive: https://www.dataarchive.lissdata.nl

Questions vx22a034 - vx22a047 and vx22a048 - vx22a061 concern the healthcare providers with whom an appointment was postponed or cancelled (vx22a026 - vx22a032). If more than two healthcare providers were chosen under vx22a026 - vx22a032, these questions concerned a random two of those healthcare providers (healthcare providers from categories other than "a different healthcare provider" were given priority). The random choice was based on variables vx22a001 - vx22a006. For example, if a respondent chose option 5 (home care) in questions vx22a026 - vx22a032 and vx22a001 = 5, then vx22a021 is "home care". For the second healthcare provider, the subsequent permutation variables were used.

vx22a001 - vx22a006

Permutation variables for choices vx22a021 and vx22a022

1. vx22a021/vx22a022=GP or GP's practice nurse
2. vx22a021/vx22a022=dentist or dental hygienist
3. vx22a021/vx22a022=physiotherapist, dietician or remedial therapist
4. vx22a021/vx22a022=hospital specialist
5. vx22a021/vx22a022=domiciliary carer
6. vx22a021/vx22a022=psychologist or psychiatrist

Questions x22a071 - vx22a095 and vx22a096 - vx22a120 concern the healthcare providers with whom an appointment was postponed or cancelled by the respondent themselves (x22a063 - vx22a069). If more than two healthcare providers were chosen under vx22a063 - vx22a069, these questions concerned a random two of those healthcare providers (healthcare providers from categories other than "a different healthcare provider" were given priority). The random choice was based on variables vx22a007 - vx22a012.

vx22a007 - vx22a012

Permutation variables for choices vx22a023 and vx22a024

1. vx22a023/vx22a024=GP or GP's practice nurse
2. vx22a023/vx22a024=dentist or dental hygienist
3. vx22a023/vx22a024=physiotherapist, dietician or remedial therapist
4. vx22a023/vx22a024=hospital specialist
5. vx22a023/vx22a024=domiciliary carer
6. vx22a023/vx22a024=psychologist or psychiatrist

vx22a013

Auxiliary variable: must vx22a034 - vx22a039 be asked?

0. No
1. Yes

vx22a014

Auxiliary variable: must vx22a048 - vx22a053 be asked?

0. No
1. Yes

vx22a015
Auxiliary variable: must vx22a071 - vx22a076 be asked?
0. No
1. Yes

vx22a016
Auxiliary variable: must vx22a096 - vx22a101 be asked?
0. No
1. Yes

vx22a017
Auxiliary variable: must vx22a040 be asked?
0. Not a dentist: ask vx22a040
1. Dentist: do not ask vx22a040

vx22a018
Auxiliary variable: must vx22a054 be asked?
0. Not a dentist: ask vx22a054
1. Dentist: do not ask vx22a054

vx22a019
Auxiliary variable: must vx22a077 be asked?
0. Not a dentist: ask vx22a077
1. Dentist: do not ask vx22a077

vx22a020
Auxiliary variable: must vx22a102 be asked?
0. Not a dentist: ask vx22a102
1. Dentist: do not ask vx22a102

vx22a021
Healthcare provider follow-up questions round one – postponed by healthcare provider
1. GP or GP's practice nurse
2. dentist or dental hygienist
3. physiotherapist, dietician or remedial therapist
4. hospital specialist
5. domiciliary carer
6. psychologist or psychiatrist
7. other healthcare provider (vx22a033)

vx22a022

Healthcare provider follow-up questions round two – postponed by healthcare provider

1. GP or GP's practice nurse
2. dentist or dental hygienist
3. physiotherapist, dietician or remedial therapist
4. hospital specialist
5. domiciliary carer
6. psychologist or psychiatrist
7. other healthcare provider (vx22a033)

vx22a023

Healthcare provider follow-up questions round one – postponed by respondent

1. GP or GP's practice nurse
2. dentist or dental hygienist
3. physiotherapist, dietician or remedial therapist
4. hospital specialist
5. domiciliary carer
6. psychologist or psychiatrist
7. other healthcare provider (vx22a070)

vx22a024

Healthcare provider follow-up questions round two – postponed by respondent

1. GP or GP's practice nurse
2. dentist or dental hygienist
3. physiotherapist, dietician or remedial therapist
4. hospital specialist
5. domiciliary carer
6. psychologist or psychiatrist
7. other healthcare provider (vx22a070)

Page 1

all respondents
{intro}
Reason for this study

This study is conducted on behalf of RIVM (the Dutch National Institute of Public Health and Environmental Protection) and concerns postponed medical care during the COVID-19 pandemic. RIVM wishes to know more about, for example, which forms of healthcare that you required were postponed, why these postponements occurred, and whether these postponements had any consequences for your health.

The researchers want to learn from the experiences of people during the pandemic so that they can take these into account in the event of future outbreaks of diseases.

Answer type: None

Page 2

all respondents
{intro2}
What is postponed care?

By "postponed care" we mean all healthcare that took place at a later date than usual as a result of the COVID-19 pandemic. This includes appointments with GPs, dentists, physiotherapists, specialists, domiciliary carers, psychologists and hospital treatments, for example.

It also covers both appointments that you personally postponed, appointments that you chose not to make at all, and appointments that were postponed or cancelled by the healthcare provider in question.
Below are a few examples.

Because of the COVID-19 pandemic:

- an appointment with a specialist at an outpatient clinic was postponed;
- you refrained from making an appointment with your GP;
- you wanted to schedule an appointment with your GP, but it was not possible to do so;
- you postponed an appointment with your dentist or dental hygienist;
- an appointment for surgery or day treatment was cancelled;
- an appointment with your physiotherapist, psychologist or for care in a private clinic was cancelled or postponed;
- an appointment with your domiciliary carer was cancelled or postponed;
- appointments with other healthcare providers were cancelled.

Answer type: None

Page 3

all respondents
{intro_zorgverlener}
The following questions concern appointments that were postponed or cancelled **by the healthcare provider**. Questions about appointments that were cancelled by you will be asked later on in this questionnaire.

Answer type: None

Page 4

all respondents
vx22a025
When we talk about "the COVID-19 period", we mean the period from the start of the COVID-19 crisis in March 2020 up to now.

During the COVID-19 period, have one or more of your scheduled medical appointments or treatments been postponed or cancelled **by the healthcare provider**?

Answer type: Radio buttons
Categories:

1. Yes
2. No
3. Not applicable, I did not have any scheduled appointments

Page 5

if (vx22a025 = 1)
vx22a026 - vx22a032
Which healthcare provider postponed or cancelled an appointment or treatment?
Multiple answers possible.

Answer type: Checkboxes
Categories:

vx22a026 my GP or my GP's practice nurse
vx22a027 my dentist or dental hygienist
vx22a028 my physiotherapist, dietician or remedial therapist
vx22a029 a hospital specialist
vx22a030 a domiciliary carer
vx22a031 my psychologist or psychiatrist

vx22a032 a different healthcare provider, namely:

0. No
1. Yes

vx22a033
Question type: Inline text field attached to code 1 of question "vx22a032"
Answer type: String

Page 6

if (vx22a013 = 1)
vx22a034 - vx22a039
You indicated that an appointment or treatment with **a hospital specialist** was postponed or cancelled.

What kind of appointment was it?
Multiple answers possible.

Answer type: Checkboxes
Categories:

vx22a034 Surgery
vx22a035 Day treatment
vx22a036 Diagnosis
vx22a037 Checkup at outpatient clinic
vx22a038 Other appointment at outpatient clinic
vx22a039 I don't know

0. No
1. Yes

Page 7

if (vx22a017 = 0)
vx22a040
You indicated that an appointment with a **[vx22a021]** was postponed or cancelled.

Did this concern postponed care for a chronic disease?

A chronic disease is a condition that will never completely go away, such as arthritis, asthma, COPD, diabetes and cardiovascular disease.

Answer type: Radio buttons
Categories:

1. Yes

2. No

Page 8

if (vx22a025 = 1)
vx22a041
How often was an appointment with a **[vx22a021]** postponed or cancelled during the COVID-19 period?

Answer type: Radio buttons
Categories:
1. 1 time
2. 2 times
3. 3 times or more

Page 9

if (vx22a025 = 1)
vx22a042 – vx22a043
You indicated that an appointment with a **[vx22a021]** was postponed or cancelled.

When did this happen, approximately?

If you do not know, you can provide an estimate. If it happened several times, base your answer on the appointment for which the postponement/cancellation was the most inconvenient for you.

Question type: Mixed table
Sub-questions:
vx22a042
Answer type: Dropdown
Label-left: Month:
Categories:
1. January
2. February
3. March
4. April
5. May
6. June
7. July
8. August
9. September
10. October

11. November

12. December

vx22a043
Answer type: Dropdown
Label-left: Year:
Categories:

2020. 2020

2021. 2021

2022. 2022

Page 10

if (vx22a025 = 1)
vx22a044
You indicated that an appointment with a **[vx22a021]** was postponed or cancelled.

Did the postponement of that appointment have consequences for your health?
E.g. complaints, increased complaints, stress.

Answer type: Radio buttons
Categories:

1. Yes, a lot

2. Yes, a bit

3. No

Page 11

if (vx22a044 < 3)
vx22a045
You indicated that an appointment with a **[vx22a021]** was postponed or cancelled.

What grade would you give your health at the time when you experienced the greatest number of negative consequences of the postponement of the appointment?

Answer type: Radio buttons
Layout: horizontal
Categories:
1. 1 Very poor
2. 2
3. 3
4. 4
5. 5
6. 6
7. 7
8. 8
9. 9
10. 10 Perfect

Page 12

if (vx22a044 < 3)
vx22a046
You indicated that an appointment with a **[vx22a021]** was postponed or cancelled.

Are you still experiencing negative consequences as a result of the postponement of that appointment?
E.g. complaints, increased complaints, stress.

Answer type: Radio buttons
Categories:
1. Yes, a lot
2. Yes, a bit
3. No

Page 13

if (vx22a025 = 1)
vx22a047
You indicated that an appointment with a **[vx22a021]** was postponed or cancelled.

What happened with regard to the appointment?

Answer type: Radio buttons
Categories:

1. It took place at a later date, as originally intended
2. The appointment took place at a later date, but with a different care provider or organization
3. The appointment took place in a different form, e.g. by telephone or video call
4. A new appointment was scheduled, but that appointment has not yet taken place
5. No new appointment has been made (yet)
6. Because my health was deteriorating, I had to make use of emergency care
7. The appointment is no longer necessary

Page 14

if (vx22a014 = 1)
vx22a048 - vx22a053
You indicated that an appointment or treatment with **a hospital specialist** was postponed or cancelled.

What kind of appointment was it?
Multiple answers possible.

Answer type: Checkboxes
Categories:

vx22a048 Surgery
vx22a049 Day treatment
vx22a050 Diagnosis
vx22a051 Checkup at outpatient clinic
vx22a052 Other appointment at outpatient clinic
vx22a053 I don't know

0. No
1. Yes

Page 15

if (vx22a018 = 0)
vx22a054
You indicated that an appointment with a **[vx22a022]** was postponed or cancelled.

Did this concern postponed care for a chronic disease?

A chronic disease is a condition that will never completely go away, such as arthritis, asthma, COPD, diabetes and cardiovascular disease.

Answer type: Radio buttons
Categories:
1. Yes
2. No

Page 16

if (count(vx22a026 - vx22a032) > 1)
vx22a055
How often was an appointment with a **[vx22a022]** postponed or cancelled during the COVID-19 period?

Answer type: Radio buttons
Categories:
1. 1 time
2. 2 times
3. 3 times or more

Page 17

if (count(vx22a026 - vx22a032) > 1)
vx22a056 – vx22a057
You indicated that an appointment with a **[vx22a022]** was postponed or cancelled.

When did this happen, approximately?

If you do not know, you can provide an estimate. If it happened several times, base your answer on the appointment for which the postponement/cancellation was the most inconvenient for you.

Question type: Mixed table
Sub-questions:
vx22a056

Answer type: Dropdown
Label-left: Month:
Categories:

1. January

2. February

3. March

4. April

5. May

6. June

7. July

8. August

9. September

10. October

11. November

12. December

vx22a057
Answer type: Dropdown
Label-left: Year
Categories:

2020. 2020

2021. 2021

2022. 2022

Page 18

if (count(vx22a026 - vx22a032) > 1)
vx22a058
You indicated that an appointment with a **[vx22a022]** was postponed or cancelled.

Did the postponement of that appointment have consequences for your health?
E.g. complaints, increased complaints, stress.

Answer type: Radio buttons
Categories:

1. Yes, a lot

2. Yes, a bit

3. No

Page 19

if (vx22a058 < 3)
vx22a059
You indicated that an appointment with a **[vx22a022]** was postponed or cancelled.

What grade would you give your health at the time when you experienced the greatest number of negative consequences of the postponement of the appointment?

Answer type: Radio buttons
Layout: horizontal
Categories:

1. 1 Very poor
2. 2
3. 3
4. 4
5. 5
6. 6
7. 7
8. 8
9. 9
10. 10 Perfect

Page 20

if (vx22a058 < 3)
vx22a060
You indicated that an appointment with a **[vx22a022]** was postponed or cancelled.

Are you still experiencing negative consequences as a result of the postponement of that appointment?
E.g. complaints, increased complaints, stress.

Answer type: Radio buttons
Categories:

1. Yes, a lot
2. Yes, a bit
3. No

Page 21

if (count(vx22a026 - vx22a032) > 1)
vx22a061
You indicated that an appointment with a **[vx22a022]** was postponed or cancelled.

What happened with regard to the appointment?

Answer type: Radio buttons
Categories:
1. It took place at a later date, as originally intended
2. The appointment took place at a later date, but with a different care provider or organization
3. The appointment took place in a different form, e.g. by telephone or video call
4. A new appointment was scheduled, but that appointment has not yet taken place
5. No new appointment has been made (yet)
6. Because my health was deteriorating, I had to make use of emergency care
7. The appointment is no longer necessary

Page 22

all respondents
{intro_zelf}
The following questions are about postponed care or care that **you personally postponed**. For example because **you** did not make an appointment or because **you** personally cancelled or postponed the appointment.

Answer type: None

Page 23

all respondents
vx22a062
These questions still concern "the COVID-19 period", i.e. the period from the start of the COVID-19 crisis in March 2020 up to now.

At any point, did **you** personally choose not to make an appointment because of the COVID-19 pandemic, even though you actually needed healthcare? Or did **you personally** postpone an appointment during the COVID-19 pandemic?

Scheduled appointments with, for example, your dentist or dental hygienist also count for the purposes of this question.

This question does not concern healthcare needed due to the COVID-19 virus itself, but healthcare for other health problems.

Answer type: Radio buttons
Categories:
1. Yes
2. No
3. Not applicable, I did not need any care

Page 24

if (vx22a062 = 1)
vx22a063 - vx22a069
Which care provider did you postpone an appointment with or which care provider did you choose not to make an appointment with even though you actually needed to?
Multiple answers possible.

Answer type: Checkboxes
Categories:
vx22a063 my GP or my GP’s practice nurse
vx22a064 my dentist or dental hygienist
vx22a065 my physiotherapist, dietician or remedial therapist
vx22a066 a hospital specialist
vx22a067 a domiciliary carer
vx22a068 my psychologist or psychiatrist
vx22a069 a different healthcare provider, namely:
0. No
1. Yes

vx22a070
Question type: Inline text field attached to code 1 of question "vx22a069"
Answer type: String

Page 25

if (vx22a015 = 1)
vx22a071 - vx22a076
You indicated that you postponed or cancelled an appointment or treatment with **a hospital specialist**.

What kind of appointment was it?
Multiple answers possible.

Answer type: Checkboxes
Categories:

vx22a071 Surgery

vx22a072 Day treatment

vx22a073 Diagnosis

vx22a074 Checkup at outpatient clinic

vx22a075 Other appointment at outpatient clinic

vx22a076 I don't know

0. No
1. Yes

Page 26

if (vx22a019 = 0)
vx22a077
You indicated that you postponed or cancelled an appointment or treatment with a **[vx22a023]**.

Was this an appointment relating to a chronic disease?

A chronic disease is a condition that will never completely go away, such as arthritis, asthma, COPD, diabetes and cardiovascular disease.

Answer type: Radio buttons
Categories:

1. Yes

2. No

Page 27

if (vx22a062 = 1)
vx22a078 - vx22a087
You indicated that you postponed or cancelled an appointment or treatment with a **[vx22a023]**.

For what reason or reasons did you cancel or postpone your appointment or not contact the **[vx22a023]** at all?
Multiple answers possible.

Answer type: Checkboxes
Categories:

vx22a078 I was afraid of being infected

vx22a079 I was afraid of infecting others with COVID-19

vx22a080 I did not want to increase the burden on the healthcare provider

vx22a081 I was afraid that the healthcare provider would not have time

vx22a082 Because of the COVID-19 crisis, I did not have enough money to pay for healthcare

vx22a083 Because of the measures against COVID-19, making an appointment was too complicated

vx22a084 I did not know whether appointments with the healthcare provider were allowed again

vx22a085 I felt that my issue was not urgent enough

vx22a086 The healthcare provider was difficult to reach

vx22a087 Other reason, namely:

0. No
1. Yes

vx22a088
Question type: Inline text field attached to code 1 of question "vx22a087"
Answer type: String

Page 28

if (vx22a062 = 1)
vx22a089
You indicated that you postponed or cancelled an appointment or treatment with a **[vx22a023]**.

How often did you postpone or cancel appointments during the COVID-19 period?

Answer type: Radio buttons
Categories:

1. 1 time

2. 2 times

3. 3 times or more

Page 29

if (vx22a062 = 1)
vx22a090 – vx22a091
You indicated that you postponed or cancelled an appointment or treatment with a **[vx22a023]**.

When did this happen, approximately?
If you do not know, you can provide an estimate. If it happened several times, base your answer on the appointment for which the postponement/cancellation was the most inconvenient for you.

Question type: Mixed table
Sub-questions:
vx22a090

Answer type: Dropdown
Label-left: Month:
Categories:

1. January
2. February
3. March
4. April
5. May
6. June
7. July
8. August
9. September
10. October
11. November
12. December

vx22a091
Answer type: Dropdown
Label-left: Year:
Categories:

2020. 2020
2021. 2021
2022. 2022

Page 30

if (vx22a062 = 1)
vx22a092
You indicated that you postponed or cancelled an appointment or treatment with a **[vx22a023]**.

Did the postponement of that appointment have consequences for your health?
E.g. complaints, increased complaints, stress.

Answer type: Radio buttons
Categories:

1. Yes, a lot
2. Yes, a bit
3. No

Page 31

if (vx22a092 < 3)
vx22a093
You indicated that you postponed or cancelled an appointment or treatment with a **[vx22a023]**.

What grade would you give your health at the time when you experienced the greatest number of negative consequences of the postponement of the appointment?

Answer type: Radio buttons
Layout: horizontal
Categories:

1. 1 Very poor
2. 2
3. 3
4. 4
5. 5
6. 6
7. 7
8. 8
9. 9
10. 10 Perfect

Page 32

if (vx22a092 < 3)
vx22a094
You indicated that you postponed or cancelled an appointment or treatment with a **[vx22a023]**.

Are you still experiencing any negative consequences because of the postponement of this healthcare?
E.g. complaints, increased complaints, stress.

Answer type: Radio buttons
Categories:

1. Yes, a lot
2. Yes, a bit
3. No

Page 33

if (vx22a062 = 1)
vx22a095
You indicated that you postponed or cancelled an appointment or treatment with a **[vx22a023]**.

Have you received that healthcare since then?

Answer type: Radio buttons
Categories:
1. Yes, fully
2. Yes, partially
3. No, and I am still in need of care
4. No, but I no longer need care (for example because the complaint went away by itself)

Page 34

if (vx22a016 = 1)
vx22a096 - vx22a101
You indicated that you postponed or cancelled an appointment or treatment with **a hospital specialist**.

What kind of appointment was it?
Multiple answers possible.

Answer type: Checkboxes
Categories:
vx22a096 Surgery
vx22a097 Day treatment
vx22a098 Diagnosis
vx22a099 Checkup at outpatient clinic
vx22a100 Other appointment at outpatient clinic
vx22a101 I don’t know
0. No
1. Yes

Page 35

if (vx22a020 = 0)
vx22a102
You indicated that you postponed or cancelled an appointment or treatment with a **[vx22a024]**.

Was this an appointment relating to a chronic disease?

A chronic disease is a condition that will never completely go away, such as arthritis, asthma, COPD, diabetes and cardiovascular disease.

Answer type: Radio buttons
Categories:
1. Yes
2. No

Page 36

if (count(vx22a063 - vx22a069) > 1)
vx22a103 - vx22a112
You indicated that you postponed or cancelled an appointment or treatment with a **[vx22a024]**.

For what reason or reasons did you cancel or postpone your appointment or not contact the **[vx22a024]** at all?
Multiple answers possible.

Answer type: Checkboxes
Categories:
vx22a103 I was afraid of being infected
vx22a104 I was afraid of infecting others with COVID-19
vx22a105 I did not want to increase the burden on the healthcare provider
vx22a106 I was afraid that the healthcare provider would not have time
vx22a107 Because of the COVID-19 crisis, I did not have enough money to pay for healthcare
vx22a108 Because of the measures against COVID-19, making an appointment was too complicated
vx22a109 I did not know whether appointments with the healthcare provider were allowed again
vx22a110 I felt that my issue was not urgent enough
vx22a111 The healthcare provider was difficult to reach
vx22a112 Other reason, namely:
0. No
1. Yes

vx22a113
Question type: Inline text field attached to code 1 of question "vx22a112"
Answer type: String

Page 37

if (count(vx22a063 - vx22a069) > 1)
vx22a114
You indicated that you postponed or cancelled an appointment or treatment with a **[vx22a024]**.

How often did you postpone or cancel appointments during the COVID-19 period?

Answer type: Radio buttons
Categories:
1. 1 time
2. 2 times
3. 3 times or more

Page 38

if (count(vx22a063 - vx22a069) > 1)
vx22a115 – vx22a116
You indicated that you postponed or cancelled an appointment or treatment with a **[vx22a024]**.

When did this happen, approximately?
If you do not know, you can provide an estimate. If it happened several times, base your answer on the appointment for which the postponement/cancellation was the most inconvenient for you.

Question type: Mixed table
Sub-questions:
vx22a115
Answer type: Dropdown
Label-left: Month:
Categories:
1. January
2. February
3. March
4. April
5. May
6. June
7. July
8. August
9. September

10. October

11. November

12. December

vx22a116
Answer type: Dropdown
Label-left: Year:
Categories:

2020. 2020

2021. 2021

2022. 2022

Page 39

if (count(vx22a063 - vx22a069) > 1)
vx22a117
You indicated that you postponed or cancelled an appointment or treatment with a **[vx22a024]**.

Did the postponement of that appointment have consequences for your health?
E.g. complaints, increased complaints, stress.

Answer type: Radio buttons
Categories:

1. Yes, a lot

2. Yes, a bit

3. No

Page 40

if (vx22a117 < 3)
vx22a118
You indicated that you postponed or cancelled an appointment or treatment with a **[vx22a024]**.

What grade would you give your health at the time when you experienced the greatest number of negative consequences of the postponement/cancellation of the appointment?

Answer type: Radio buttons
Layout: horizontal
Categories:

1. 1 Very poor

2. 2

3. 3

4. 4

5. 5
6. 6
7. 7
8. 8
9. 9
10. 10 Perfect

Page 41

if (vx22a117 < 3)
vx22a119
You indicated that you postponed or cancelled an appointment or treatment with a **[vx22a024]**.

Are you still experiencing any negative consequences because of the postponement of this healthcare?
E.g. complaints, increased complaints, stress.

Answer type: Radio buttons
Categories:
1. Yes, a lot
2. Yes, a bit
3. No

Page 42

if (count(vx22a063 - vx22a069) > 1)
vx22a120
You indicated that you postponed or cancelled an appointment or treatment with a **[vx22a024]**.

Have you since received that healthcare?

Answer type: Radio buttons
Categories:
1. Yes, fully
2. Yes, partially
3. No, and I am still in need of the care in question
4. No, but I no longer need the care in question (for example because the complaint went away by itself)

Page 43

all respondents

vx22a121

What grade would you give your health right now?

Answer type: Radio buttons

Layout: horizontal

Categories:

1. 1 Very poor
2. 2
3. 3
4. 4
5. 5
6. 6
7. 7
8. 8
9. 9
10. 10 Perfect

Page 44

all respondents

vx22a122

Compared to the period immediately before the start of the COVID-19 pandemic, my health right now is...

Answer type: Radio buttons

Categories:

1. A lot worse
2. Worse
3. The same
4. Better
5. A lot better

Page 45

all respondents

vx22a123 - vx22a127

We will now present a few statements about how you experienced your health during the COVID-19 pandemic. When we talk about postponed healthcare, we mean healthcare postponed by the healthcare provider as well as healthcare postponed by you personally.

Question type: Table
Answer type: Radio buttons
Sub-questions:
vx22a123 Due to postponed healthcare during the COVID-19 pandemic, my health temporarily got worse.
vx22a124 Due to postponed healthcare during the COVID-19 pandemic, my health got permanently worse.
vx22a125 Generally speaking, I am satisfied with the healthcare I received during the COVID-19 pandemic.
vx22a126 Due to postponed healthcare during the COVID-19 pandemic, I was concerned about my health.
vx22a127 Due to postponed healthcare, I used more medication.
Categories:

1. Completely disagree
2. Disagree
3. Neutral
4. Agree
5. Completely agree

99. N/A

Page 46

all respondents

{outro}

This is the end of the questionnaire. Thank you for your participation. The results of this questionnaire will probably be presented on the website for LISS panel members and the RIVM web page on the health consequences of postponed care during the COVID-19 pandemic towards the end of this year.

You will now be asked a final few question about how you experienced this questionnaire.

Answer type: None

all respondents
vx22a128 – vx22a132
Note: Please complete the questionnaire until you are returned to the starting screen. Only then will the system register the questionnaire as **fully** completed.

Finally; what did you think of this questionnaire?

Question type: Table
Answer type: Radio buttons
Sub-questions:
vx22a128 Was it difficult to answer the questions?
vx22a129 Were the questions sufficiently clear?
vx22a130 Did the questionnaire get you thinking about things?
vx22a131 Was it an interesting subject?
vx22a132 Did you enjoy answering the questions?
Categories:
1. 1 Certainly not
2. 2
3. 3
4. 4
5. 5 Certainly yes

---

vx22a133
Starting date questionnaire
string

vx22a134
Starting time questionnaire
string

vx22a135
End date questionnaire
string

vx22a136
End time questionnaire
string

calculated variable
vx22a137
Duration in seconds
integer

Centerdata
Postbus 90153
5000 LE Tilburg

www.centerdata.nl
