# Supplementary material for: Postponed healthcare in The Netherlands during the COVID-19 pandemic and its impact on self-reported health
Source: Front Health Serv. 2023 Jun 23;3:1181532. doi: 10.3389/frhs.2023.1181532 (PMC10326615; doi:10.3389/frhs.2023.1181532)
Supplement: Supplementary file 2 [file Datasheet2.docx]

Supplementary Material

Postponed healthcare in the Netherlands during the COVID-19 pandemic and its impact on self-reported health

Kirsten Visscher ^‡*^, Lisanne Kouwenberg^‡^, Marije Oosterhoff , Adriënne Rotteveel, G. Ardine de Wit

^‡^ These authors contributed equally to this work and share first authorship

*** Correspondence:**Kirsten Visscher
kirsten.visscher@rivm.nl

# Supplementary Figures and Tables

Appendix 2. Representativeness of study sample in comparison with Dutch population

Table A2.1. Characteristics of the study sample in comparison with the Dutch population

| **Variable** | **Category** | **Study sample (n=2.043)** | **Statistics Netherlands 2021*** |
| --- | --- | --- | --- |
| **Age** | 16-24 years | 13.0% | 13.4% |
|  | 25-34 years | 15.5% | 15.5% |
|  | 35-44 years | 14.0% | 14.3% |
|  | 45-54 years | 16.4% | 16.6% |
|  | 55-64 years | 17.0% | 16.5% |
|  | 65 + | 24.1% | 23.7% |
| **Sex** | Female | 54.7% | 50,3% |
|  | Male | 45.3% | 49,7% |
| **Education** | Primary education | 7.1% | 8.7% |
|  | Lower secondary education | 17.0% | 19.6% |
|  | Secondary vocational education | 24.4% | 26.5% |
|  | Higher secondary education | 11.9% | 9.4% |
|  | Higher professional education | 25.4% | 21.5% |
|  | University education | 14.2% | 12.7% |
|  | Don’t know / missing | **/** | 1.6% |

*Source: CBS Statline https://opendata.cbs.nl/statline/#/CBS/nl/

**Appendix 3.** Reasons to postpone healthcare on own behalf. Multiple answers possible

**Appendix 4.** Types of healthcare for which respondents experienced postponement, either postponement by the healthcare provider (n=384) or on their own initiative (n=352). Proportions do not sum up to 100%, because participants could select more than one type of healthcare.*

*107 respondents are included in both figures, since they experienced both postponement by the healthcare provider and postponed healthcare themselves.

**Appendix 5. Sensitivity analysis: self-reported health before the pandemic**

Table A5.1. Logistic regression of experiencing postponed healthcare with pre-pandemic self-reported health

|  |  | **Postponed healthcare (OR, 95% CI)** | | | |
| --- | --- | --- | --- | --- | --- |
| Variable | Category | **Full model^a^** | | **Final model^b^** | |
| Age | 15 – 24 years | ref |  |  |  |
|  | 25 – 34 years | 0.88 (0.51 – 1.51) | 0.64 |  |  |
|  | 35 – 44 years | 1.36 (0.80 – 2.31) | 0.25 |  |  |
|  | 45 – 54 years | 0.98 (0.59 – 1.62) | 0.93 |  |  |
|  | 55 – 64 years | 0.88 (0.54 – 1.45) | 0.62 |  |  |
|  | 65 years and older | 0.81 (0.49 – 1.310 | 0.38 |  |  |
| Self-reported sex | Female | 1.60* (1.26 – 2.05) | 0.00 | 1.56* (1.25 – 1.94) | 0.00 |
|  | Male | ref |  | ref |  |
| Education level | Low | 0.67* (0.47 – 0.95) | 0.03 | 0.60* (0.45 – 0.81) | 0.00 |
|  | Intermediate | 0.97 (0.74 – 1.27) | 0.82 | 0.89 (0.70 – 1.14) | 0.36 |
|  | High | ref |  |  |  |
| Urbanicity level | Urbanized | 1.15 (0.92 – 1.44) | 0.22 |  |  |
|  | Not urbanized | ref |  |  |  |
| Income level | €0 - €1.249 | 0.94 (0.64 – 1.38) | 0.75 |  |  |
|  | €1.250 - €2.399 | 0.74 (0.52 – 1.06) | 0.10 |  |  |
|  | €2.400 - €3.499 | 0.90 (0.65 – 1.24) | 0.51 |  |  |
|  | ≥€3.500 | ref |  |  |  |
| Chronic disease | Yes | 1.74* (1.35 – 2.26) | 0.00 | 1.62* (1.28 – 2.06) | 0.00 |
|  | No | ref |  | ref |  |
| Self-reported health before the pandemic^‡^ | Poor or moderate | 1.88* (1.39 – 2.54) | 0.00 | 1.86* (1.39 – 2.47) | 0.00 |
|  | Good, very good, excellent | ref |  | ref |  |

CI = confidence interval, n = number, OR = odds ratio, ref = reference category ^a^ n=1,542, constant: beta = 0.30, tolerance > 0.7 and VIF < 1.4 ^b^n=1,634, constant: beta = 0.17, tolerance > 0.8 and VIF < 1.2
*p < 0.05
^‡^ Self-reported before the pandemic was categorized due to low numbers per category.

Table A5.2. Logistic regression of experiencing negative health consequences with pre-pandemic self-reported health

|  |  | **Negative consequences (OR, 95% CI)** | | | |
| --- | --- | --- | --- | --- | --- |
| Variable | Category | **Full model^a^** | | **Final model^b^** | |
| Age | 15 – 24 years | ref |  |  |  |
|  | 25 – 34 years | 2.85* (1.13 – 7.18) | 0.03 |  |  |
|  | 35 – 44 years | 1.55 (0.62 – 3.87) | 0.34 |  |  |
|  | 45 – 54 years | 1.36 (0.55 – 3.38) | 0.50 |  |  |
|  | 55 – 64 years | 0.62 (0.26 – 1.47) | 0.28 |  |  |
|  | 65 years and older | 0.85 (0.36 – 2.01) | 0.71 |  |  |
| Self-reported sex | Female | 0.87 (0.35 – 1.35) | 0.87 |  |  |
|  | Male | ref |  |  |  |
| Education level | Low | 1.66 (0.87 – 3.16) | 0.12 |  |  |
|  | Intermediate | 1.53 (0.94 – 2.50) | 0.09 |  |  |
|  | High | ref |  |  |  |
| Urbanicity level | Urbanized | 0.99 (0.67 – 1.48) | 0.97 |  |  |
|  | Not urbanized | ref |  |  |  |
| Income level | €0 - €1.249 | 2.02 (0.99 – 4.12) | 0.05 |  |  |
|  | €1.250 - €2.399 | 1.21 (0.65 – 2.26) | 0.54 |  |  |
|  | €2.400 - €3.499 | 1.05 (0.58 – 1.90) | 0.86 |  |  |
|  | ≥€3.500 | ref |  |  |  |
| Chronic disease | Yes | 2.16* (1.36 – 3.43) | 0.00 | 1.58* (1.06 -2.37) | 0.03 |
|  | No | ref |  |  |  |
| Self-reported health before the pandemic^‡^ | Poor or moderate | 1.50 (0.92 – 2.43) | 0.11 | 1.83* (1.17 – 2.84) | 0.01 |
|  | Good, very good, execellent | ref |  | ref |  |

CI = confidence interval, n = number, OR = odds ratio, ref = reference category ^a^ n = 467, Constant: beta = 0.23, tolerance > 0.7 and VIF < 1.4
^b^ n= 498, Constant: beta = 0.43, tolerance > 0.8 and VIF < 1.3
*p < 0.05
^‡^ Self-reported before the pandemic was categorized due to low numbers per category.

**Appendix 6. Sensitivity analysis: subgroup without postponed dental care**

**Table A6.1.** Logistic regression of experiencing postponed healthcare without patients who experienced postponed dental care

| **Variable** | **Category** | **Full model^a^** | | **Final model^b^** | |
| --- | --- | --- | --- | --- | --- |
|  |  | **OR (95% CI)** | **P** | **OR (95% CI)** | **P** |
| Age | 15 – 24 years | ref |  | ref |  |
|  | 25 – 34 years | 1.26 (0.78-2.02) | 0.35 |  |  |
|  | 35 – 44 years | 1.62 (1.00-2.62) | 0.05 |  |  |
|  | 45 – 54 years | 0.95 (0.59-1.53) | 0.83 |  |  |
|  | 55 – 64 years | 1.00 (0.63-1.60) | 1.00 |  |  |
|  | 65 years and older | 0.93 (0.60-1.44) | 0.74 |  |  |
| Self-reported sex | Female | 1.56* (1.23-1.98) | 0.00 | 1.59* (1.28-1.97) | 0.00 |
|  | Male | ref |  | ref |  |
| Education level | Low | 0.59* (0.42-0.84) | 0.00 | 0.54* (0.40-0.73) | 0.00 |
|  | Intermediate | 0.99 (0.75-1.29) | 0.92 | 0.87 (0.68-1.11) | 0.26 |
|  | High | ref |  | ref |  |
| Income in € | €0 - €1.249 | 1.12 (0.77-1.65) | 0.55 |  |  |
|  | €1.250 - €2.399 | 0.87 (0.61-1.24) | 0.45 |  |  |
|  | €2.400 - €3.499 | 0.92 (0.66-1.28) | 0.62 |  |  |
|  | ≥€3.500 | ref |  |  |  |
| Urbanicity level | Urbanized | 1.14 (0.91-1.42) | 0.25 |  |  |
|  | Not urbanized | ref |  |  |  |
| Chronic disease | Yes | 1.69* (1.30-2.19) | 0.00 | 1.56* (1.22-1.99) | 0.00 |
|  | No | ref |  | ref |  |
| Self-reported health during the pandemic | Poor | 2.42 (0.82 – 7.09) | 0.11 |  |  |
|  | Moderate | 3.29* (1.67 6.49) | 0.00 |  |  |
|  | Good | 1.95 (1.05 – 3.60) | 0.03 |  |  |
|  | Very good | 1.47 (0.78 2.79) | 0.23 |  |  |
|  | Excellent | ref |  |  |  |

CI = confidence interval, n = number, OR = odds ratio, ref = reference category ^a^ n = 1773, Constant: beta = 0.11, tolerance > 0.6 and VIF < 1.3
^b^ n = 1889, Constant: beta = 0.17, tolerance > 0.9 and VIF < 1.1
*p < 0.05

**Table A6.2.** Logistic regression of experiencing negative health consequences without patients who experienced postponed dental care

| **Variable** | **Category** | **Full model^a^** | | **Final model^b^** | |
| --- | --- | --- | --- | --- | --- |
|  |  | **OR (95% CI)** | **P** | **OR (95% CI)** | **P** |
| Age | 15 – 24 years | ref |  |  |  |
|  | 25 – 34 years | 1.71 (0.73-3.98) | 0.22 |  |  |
|  | 35 – 44 years | 1.97 (0.84-4.64) | 0.12 |  |  |
|  | 45 – 54 years | 1.27 (0.53-3.05) | 0.60 |  |  |
|  | 55 – 64 years | 0.71 (0.31-1.64) | 0.43 |  |  |
|  | 65 years and older | 0.87 (0.38-1.99) | 0.74 |  |  |
| Self-reported sex | Female | 0.93 | 0.74 |  |  |
|  | Male | ref |  |  |  |
| Education level | Low | 1.47 (0.75-2.89) | 0.26 |  |  |
|  | Intermediate | 1.27 (0.77-2.08) | 0.35 |  |  |
|  | High | ref |  |  |  |
| Income in € | €0 - €1.249 | 2.73* (1.30-5.70) | 0.01 |  |  |
|  | €1.250 - €2.399 | 1.64 (0.87-3.12) | 0.13 |  |  |
|  | €2.400 - €3.499 | 1.68 (0.92-3.09) | 0.09 |  |  |
|  | ≥€3.500 | ref |  |  |  |
| Urbanicity level | Urbanized | 1.04 (0.69-1.56) | 0.86 |  |  |
|  | Not urbanized | ref |  |  |  |
| Chronic disease | Yes | 1.69* (1.04-2.77) | 0.04 | 1.54* (1.06-2.24) | 0.02 |
|  | No | ref |  | ref |  |
| Self-reported health during the pandemic | Poor | 1.04 (0.13 – 8.14) | 0.97 |  |  |
|  | Moderate | 1.05 (0.28 – 3.99) | 0.94 |  |  |
|  | Good | 0.91 (0.26 – 3.21) | 0.88 |  |  |
|  | Very good | 0.68 (0.18 – 2.55) | 0.57 |  |  |
|  | Excellent | ref |  |  |  |

CI = confidence interval, n = number, OR = odds ratio, ref = reference category ^a^ n = 439, Constant: beta = 0.27, tolerance > 0.6 and VIF < 1.3
^b^ n = 475 , Constant: beta = 0.56, tolerance > 0.9 and VIF < 1.1
*p < 0.05
